# Supplementary material for: Molecular Signatures of Tumour and Its Microenvironment for Precise Quantitative Diagnosis of Oral Squamous Cell Carcinoma: An International Multi-Cohort Diagnostic Validation Study
Source: Cancers (Basel). 2022 Mar 9;14(6):1389. doi: 10.3390/cancers14061389 (PMC8945999; doi:10.3390/cancers14061389)
Supplement: Supplementary file 1 [file cancers-14-01389-s001.zip › cancers-1619395-supplementary.pdf]

cancers

MDPI

Supplementary Materials

Molecular Signatures of Tumour and its Microenvironment for Precise Quantitative Diagnosis of Oral Squamous Cell Carcinoma: An International Multi-Cohort Diagnostic Validation Study

Muy-Teck Teh[1,2,3]*, Hong Ma[2], Ying-Ying Liang[3], Monica Charlotte Solomon[4], Akhilanand Chaurasia[5], Ranjitkumar Patil[5], Satyajit Ashok Tekade[6], Deepika Mishra[7], Fatima Qadir[1], Ji-Yun Stephanie Yeung[1], Xinting Liu[1], Safa Kriuar[1], Ruoqi Zhao[1], Ahmad Waseem[1], Iain L. Hutchison[1,8,9]

Citation: Teh, M.-T.; Ma, H.; Liang, Y.-Y.; Solomon, M.C.; Chaurasia, A.; Patil, R.; Tekade, S.A.; Mishra, D.; Qadir, F.; Yeung, J.-Y.S.; et al. Molecular Signatures of Tumour and Its Microenvironment for Precise Quantitative Diagnosis of Oral Squamous Cell Carcinoma: An International Multi-Cohort Diagnostic Validation Study. *Cancers* **2022**, *14*, 1389. https://doi.org/10.3390/cancers14061389

Academic Editor: Chamindie K. Punyadeera

Received: 15 February 2022
Accepted: 4 March 2022
Published: 9 March 2022

Publisher's Note: MDPI stays neutral with regard to jurisdictional claims in published maps and institutional affiliations.

Copyright: © 2022 by the authors. Licensee MDPI, Basel, Switzerland. This article is an open access article distributed under the terms and conditions of the Creative Commons Attribution (CC BY) license (https://creativecommons.org/licenses/by/4.0/).

Table S1. – qMIDS^{V2} Gene Panel Primer Sequences.

Gene	Loci	Forward Primer	Reverse Primer	Bp[a]
HOXA7	7p15.2	GCCAATTTCCGCATCTACCC	GGTAGCGGTTGAAGTGGAAC	121
CENPA	2p23.3	CTGCACCCAGTGTTTCTGTC	GAGAGTCCCCGGTATCATCC	63
NEK2	1q32.3	CATTGGCACAGGCTCCTAC	GAGCCATAGTCAAGTTCTTTCCA	90
DNMT1	19p13.2	CGATGTGGCGTCTGTGAG	TGTCCTTGCAGGCTTTACATT	64
INHBA*	7p14.1	GCTCAGACAGCTCTTACCACA	AAATTCTCTTTCTGGTCCCCACT	69
FOXM1	12p13.33	ACTTTAAGCACATTGCCAAGC	CGTGCAGGGAAAGGTTGT	63
TOP2A*	17q21.2	CAGTGAAGAAGACAGCAGCAAA	AAGCTGGATCCCTTTTAGTTCC	96
BIRC5*	17q25.3	AGAACTGGCCCTTCTTGGA	ACACTGGGCCAAGTCTGG	104
MMP13*	11q22.2	TGAGCTGGACTCATTGTCGG	AGGTAGCGCTCTGCAAACTG	94
CXCL8*	4q13.3	AAGTTTTTGAAGAGGGCTGAGA	TGGCATCTTCACTGATTCTTGGA	74
NR3C1*	5q31.3	TCCCTGGTCGAACAGTTTTT	GCTGGATGGAGGAGAGCTTA	77
IVL	1q21.3	TGCCTGAGCAAGAATGTGAG	TTCCTCATGCTGTTCCCAGT	83
CBX7*	22q13.1	CGAGTATCTGGTGAAGTGGAAA	GGGGGTCCAAGATGTGCT	77
S100A16*	1q21.3	CAAGATCAGCAAGAGCAGCTT	GAGCTTATCCGCAGCCTTC	94
YAP1	11q22.1	ACAATGACGACCAATAGCTCAG	CCACTGTCTGTACTCTCATCTCG	77
POLR2A	17p13.1	TCCGTATTCGCATCATGAAC	TCATCCATCTTGTCCACCAC	73

*Indicates the 8 new genes in qMIDS^{V2} over qMIDS^{V1}. [a]Indicates the basepair length of each PCR amplicon.

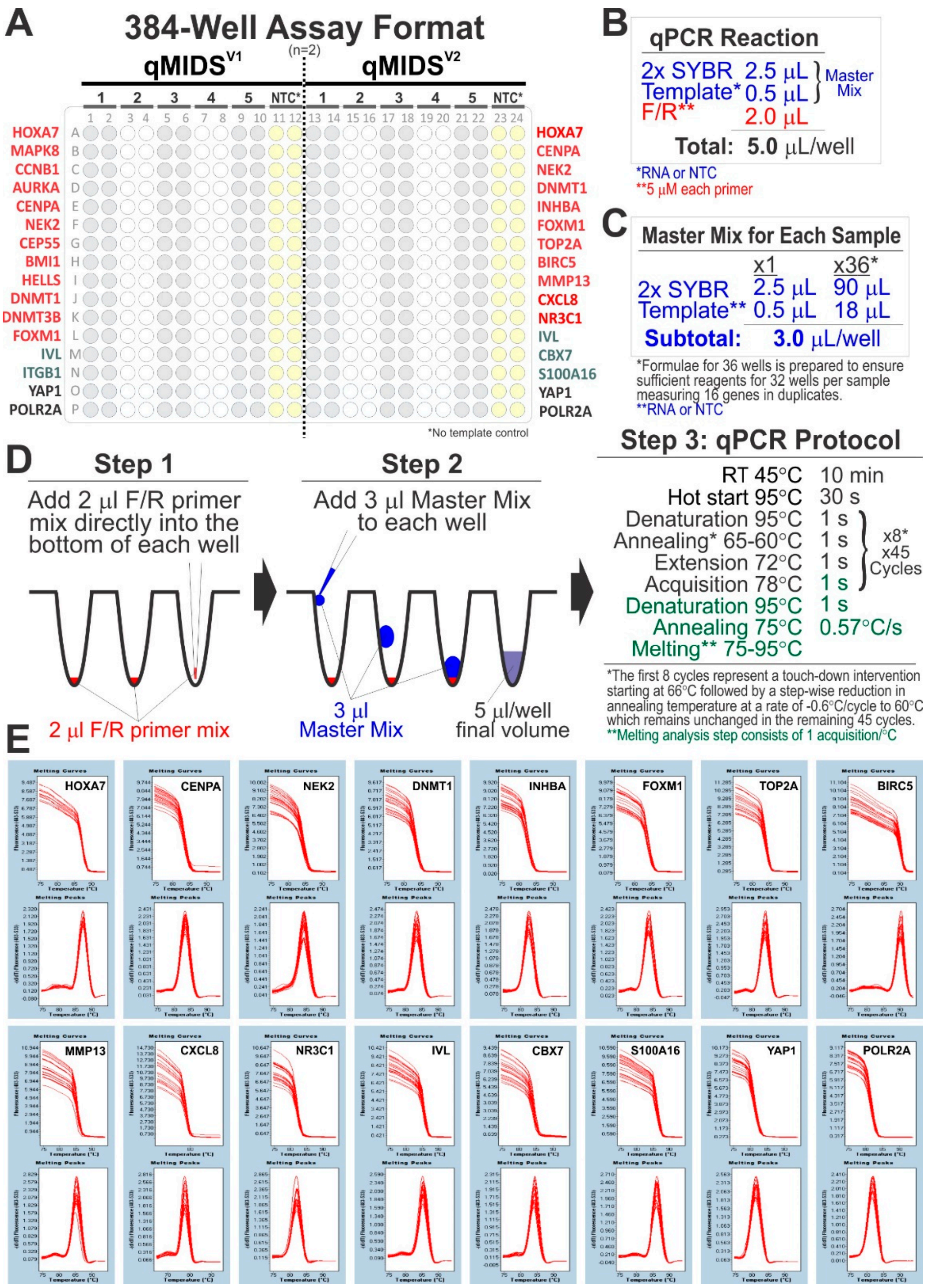

Figure S1. – qMIDS^{V1} vs qMIDS^{V2} 384-well assay format and protocols. A, qMIDS^{V1} vs qMIDS^{V2} assay layout for 5 samples in duplicates. B, qPCR reaction composition per well. C, Master mix preparation for each sample sufficient for n=32 wells. D, Primer (Step 1) and master mix (Step 2) loading procedures, and qPCR cycling protocol (Step 3). Please note that this fast-cycling protocol was achievable preferably by using qPCRBIO SyGreen 1-Step Go (PCR Biosystems, PB25.31-12) or potentially others that allow high speed qPCR cycling. For other standard SYBR green master mix reagents, increase the 1s steps to at least 5s. E, A representative melting curve analysis (top panels: y-axis, fluorescence and x-axis, temperature °C) for each of the 16 genes in qMIDS^{V2} showing single melting peak [bottom panels: y-axis, -(d/dT) fluorescence and x-axis, temperature °C] for each gene.

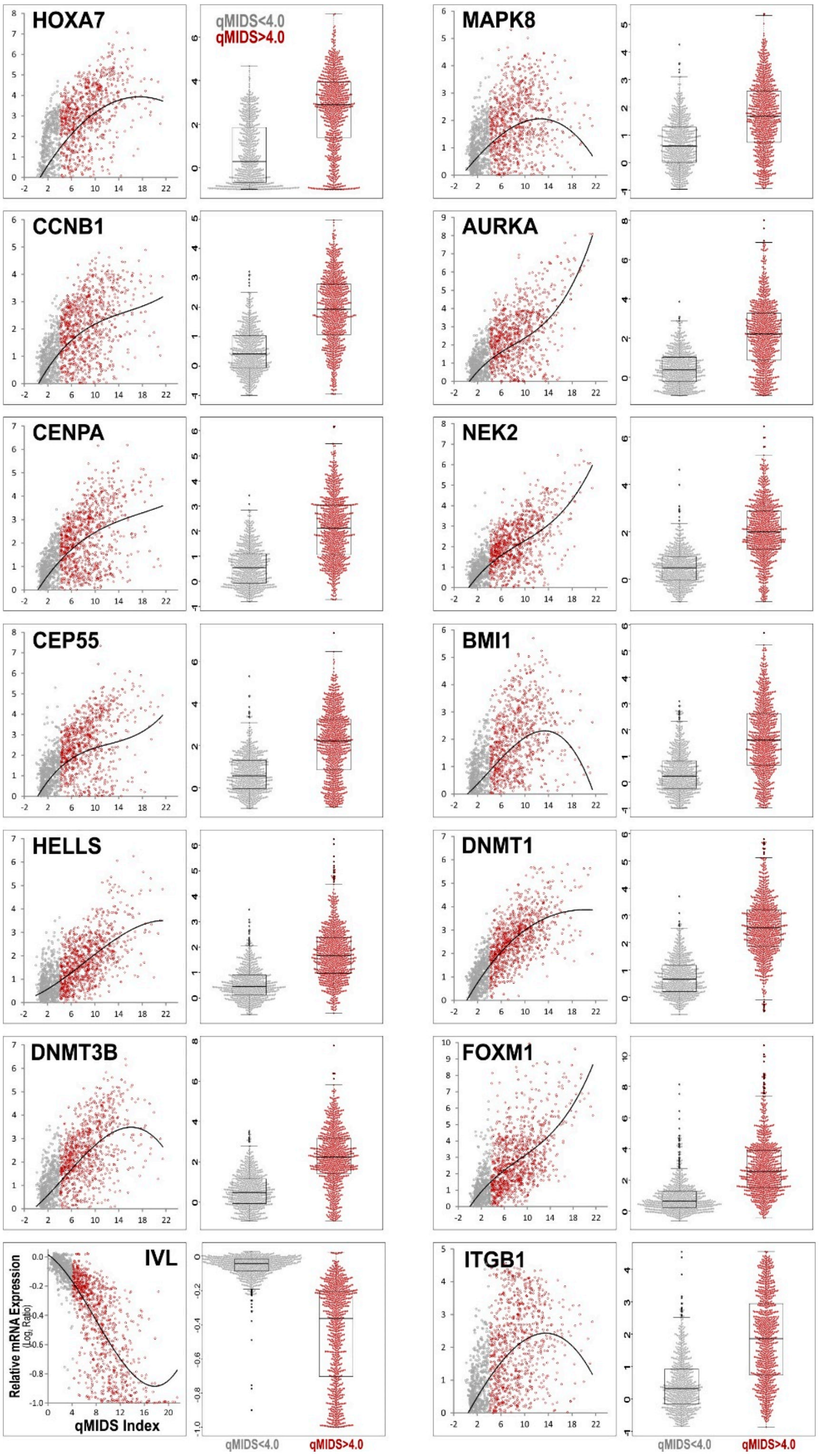

Figure S2. Individual target gene expression pattern in 1761 samples (normal/margin and core OSCC samples) in correlation with qMIDS^{V1} index values (scattered dot-plots, left panel) and segregated beeswarm plots (cut-off at 4.0 (Teh *et al*, 2013), right panel). Data points in grey and red indicate qMIDS <4.0 and >4.0, respectively. Regression R^2 and t-test P-values are shown in Supplemental information Figure S3.

A Distribution Method

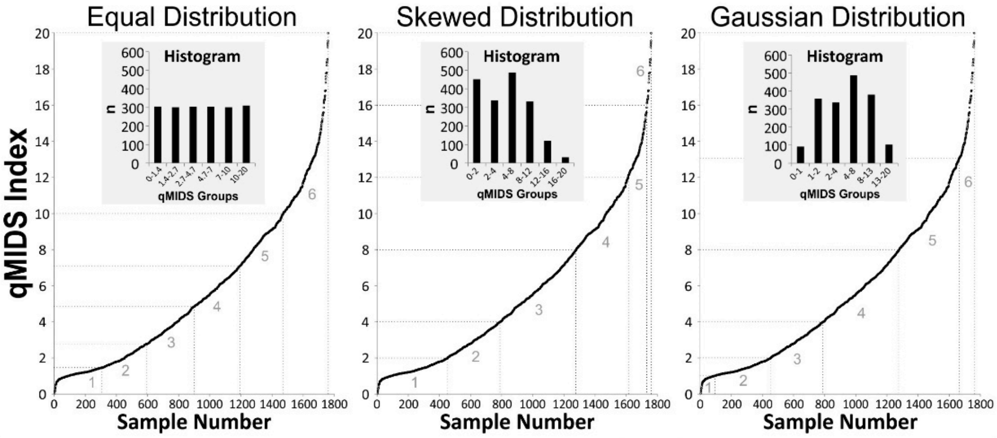

Equal	HOXA7	MAPK8	CCNB1	AURKA	CENPA	NEK2	CEP55	BMI1	HELLS	DNMT1	DNMT3B	FOXM1	IVL	ITGB1
Linear R^2	0.84	0.35	0.46	0.75	0.87	0.81	0.73	0.66	0.70	0.82	0.81	0.75	0.87	0.70
Polynomial R^2	1.30	0.74	1.29	1.37	1.43	1.65	0.93	1.04	1.58	2.22	1.00	1.58	2.31	1.12
Fold Change	1.64	0.40	0.60	0.71	0.61	0.64	0.59	0.74	0.45	0.61	0.63	0.86	1.05	0.69
**Average**	1.26	0.50	0.78	0.95	0.97	1.03	0.75	0.81	0.91	1.22	0.81	1.07	1.41	0.84

Skewed	HOXA7	MAPK8	CCNB1	AURKA	CENPA	NEK2	CEP55	BMI1	HELLS	DNMT1	DNMT3B	FOXM1	IVL	ITGB1
Linear R^2	1.08	0.00	0.81	0.56	0.98	0.66	0.69	0.08	0.54	1.02	1.18	0.61	1.13	0.78
Polynomial R^2	1.30	0.74	1.29	1.37	1.43	1.65	0.93	1.04	1.58	2.22	1.00	1.58	2.31	1.12
Fold Change	1.17	0.36	0.57	0.75	0.61	0.68	0.53	0.74	0.45	0.64	0.69	0.77	1.38	0.67
**Average**	1.18	0.37	0.89	0.89	1.01	1.00	0.72	0.62	0.86	1.29	0.95	0.99	1.61	0.86

Gaussian	HOXA7	MAPK8	CCNB1	AURKA	CENPA	NEK2	CEP55	BMI1	HELLS	DNMT1	DNMT3B	FOXM1	IVL	ITGB1
Linear R^2	0.97	0.01	0.80	0.63	0.87	0.72	0.97	0.51	0.58	0.92	0.88	0.76	0.96	0.50
Polynomial R^2	1.30	0.74	1.29	1.37	1.43	1.65	0.93	1.04	1.58	2.22	1.00	1.58	2.31	1.12
Fold Change	1.64	0.39	0.59	0.72	0.60	0.63	0.58	0.69	0.42	0.65	0.60	0.87	1.18	0.65
**Average**	1.30	0.38	0.89	0.91	0.97	1.00	0.83	0.75	0.86	1.26	0.83	1.07	1.48	0.75

	HOXA7	MAPK8	CCNB1	AURKA	CENPA	NEK2	CEP55	BMI1	HELLS	DNMT1	DNMT3B	FOXM1	IVL	ITGB1
**Average R^2**	1.25	0.41	0.86	0.92	0.98	1.01	0.76	0.73	0.88	1.26	0.86	1.04	1.50	0.82

B Threshold Method

Raw Data	HOXA7	MAPK8	CCNB1	AURKA	CENPA	NEK2	CEP55	BMI1	HELLS	DNMT1	DNMT3B	FOXM1	IVL	ITGB1
ttest	3E-118	2E-69	5E-148	2E-121	1E-152	2E-165	8E-96	2E-105	2E-134	6E-264	3E-148	8E-129	3E-215	2E-119
Fold (Median)	11.0	2.8	4.6	5.8	4.0	4.3	3.8	7.0	3.7	3.9	4.6	5.0	8.6	5.8
Fold (Mean)	4.1	2.5	3.8	4.7	3.6	4.2	3.0	4.6	3.0	3.5	3.9	3.2	7.4	4.0
**Normalised**	**HOXA7**	**MAPK8**	**CCNB1**	**AURKA**	**CENPA**	**NEK2**	**CEP55**	**BMI1**	**HELLS**	**DNMT1**	**DNMT3B**	**FOXM1**	**IVL**	**ITGB1**
ttest	0.06	0.03	0.07	0.06	0.08	0.08	0.05	0.05	0.07	0.13	0.07	0.06	0.11	0.06
Mean Fold	0.12	0.04	0.06	0.08	0.06	0.07	0.05	0.09	0.05	0.06	0.07	0.06	0.12	0.08
**Average**	8.8	3.7	7.0	7.1	6.8	7.4	5.0	7.1	6.0	9.5	7.0	6.4	11.5	6.8

C Final Selection Summary

	HOXA7	MAPK8	CCNB1	AURKA	CENPA	NEK2	CEP55	BMI1	HELLS	DNMT1	DNMT3B	FOXM1	IVL	ITGB1
Distribution Method	9.4%	3.1%	6.4%	6.9%	7.4%	7.6%	5.8%	5.5%	6.6%	9.5%	6.5%	7.8%	11.3%	6.2%
Threshold Method	8.8%	3.7%	7.0%	7.1%	6.8%	7.4%	5.0%	7.1%	6.0%	9.5%	7.0%	6.4%	11.5%	6.8%
**Average Score**	9.1%	3.4%	6.7%	7.0%	7.1%	7.5%	5.4%	6.3%	6.3%	9.5%	6.8%	7.1%	11.4%	6.5%
**Selection (>7)**	✓ HOXA7	×	×	×	✓ CENPA	✓ NEK2	×	×	×	✓ DNMT1	×	✓ FOXM1	✓ IVL	×

Figure S3. Various statistical methods used for gene selection analysis on 1761 clinical samples (from Figure S2). A, Distribution methods using either equal, skewed or Gaussian distribution for grouping samples based on their qMIDS values. Insets showed histograms of $qMIDS^{V1}$ groupings (6 groups). Linear and polynomial regression analyses were applied on each distribution method. Fold changes were also calculated between group 1-3 and group 4-6. R^2 and *t*-test *P*-values were normalised and overall average values were obtained for each gene. Heat map colour grading (from low/yellow to high/red) indicates the strength of correlation with $qMIDS^{V1}$. B, Threshold method is based on $qMIDS^{V1}$ cut-off value at 4.0 (Teh *et al*, 2013). Gene expression data were either raw (relative to reference genes) or normalised (Log_2 Ratio) values. C, Final selection summary of data from A and B. Selection were made for genes with an average score above 7%.

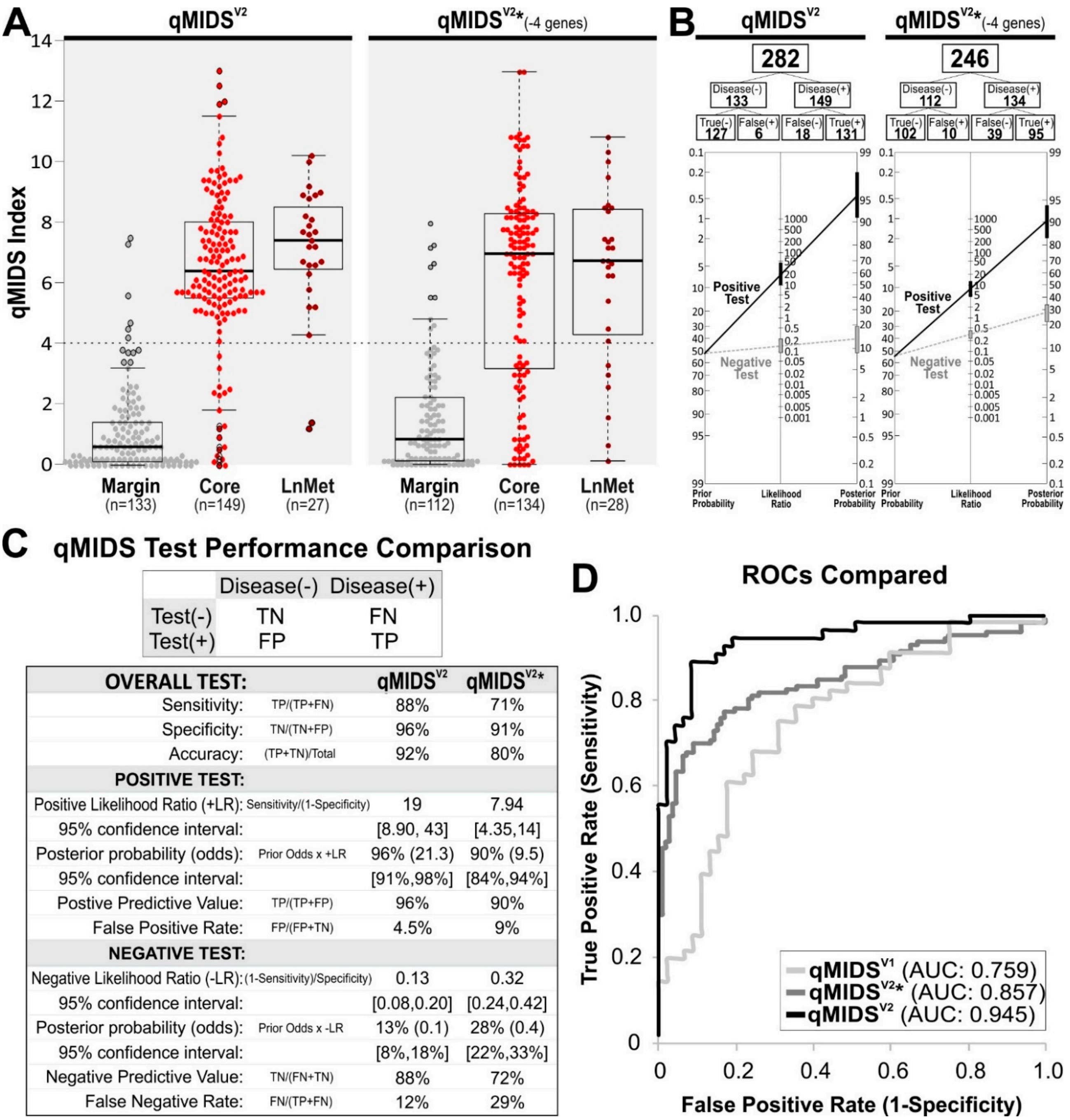

Figure S4. Diagnostic performance comparison between qMIDS^{V2} vs qMIDS^{V2*} (with 4 less effective genes removed from the panel of 14 target genes of qMIDS^{V2}). A, OSCC (margin and tumour cores) and neck lymph-node metastatic tissue samples were independently measured by either qMIDS^{V2} or qMIDS^{V2*}. The unequal sample size was due to insufficient tissue left in some samples for experimentation. B, Diagnostic performance analyses were performed on data collected from margin and tumour samples for qMIDS^{V2} or qMIDS^{V2*} from panel A. C, Diagnostic test performance table comparing between qMIDS^{V2} and qMIDS^{V2*}. D, Data from panel A were separately subjected to ROC analysis showing the comparison between qMIDS^{V1} (data taken from Figure 3D), qMIDS^{V2} and qMIDS^{V2*} with respective AUC values as shown within the panel. TN, true negative; FN, false negative; FP, false positive; TP, true positive.

A Diagnostic Test Performance Compared

	qMIDS^{V2} (qV2)	qV2-HOXA7	qV2-CENPA	qV2-NEK2	qV2-DNMT1	qV2-INHBA	qV2-FOXM1	qV2-TOP2A	qV2-BIRC5	qV2-MMP13	qV2-CXCL8	qV2-NR3C1	qV2-IVL	qV2-CBX7	qV2-S100A16
TP/(TP+FN) **Sensitivity**	88%	85%	85%	76%	83%	84%	71%	74%	81%	86%	88%	86%	85%	87%	87%
TN/(TN+FP) **Specificity**	95%	92%	93%	96%	98%	95%	96%	96%	95%	89%	89%	89%	92%	89%	71%
(TP+TN)/Total **Accuracy**	91%	88%	89%	85%	90%	89%	83%	85%	88%	88%	89%	88%	88%	88%	79%
TP/(TP+FP) **PPV**	96%	92%	93%	96%	98%	95%	95%	96%	95%	90%	90%	90%	93%	90%	77%
TN/(FN+TN) **NPV**	88%	85%	84%	78%	84%	84%	75%	77%	82%	85%	87%	85%	84%	86%	82%
FP/(FP+TN) **FPR**	5%	8%	7%	4%	2%	5%	4%	4%	5%	11%	11%	11%	8%	11%	29%
FN/(TP+FN) **FNR**	12%	15%	15%	24%	17%	16%	29%	26%	19%	14%	12%	14%	15%	13%	13%
ROC **AUC**	95%	92%	93%	94%	94%	94%	92%	93%	94%	92%	93%	93%	93%	94%	87%
**Overall Performance Score***	**10**	**7**	**7**	**6**	**9**	**8**	**5**	**5**	**7**	**6**	**7**	**6**	**7**	**7**	**3**

*Normalised Ratio of [sensitivity + specificity + accuracy + positive predictive value (PPV) + negative predictive value (NPV) + AUC] : [false positive rate (FPR) + false negative rate (FNR)]

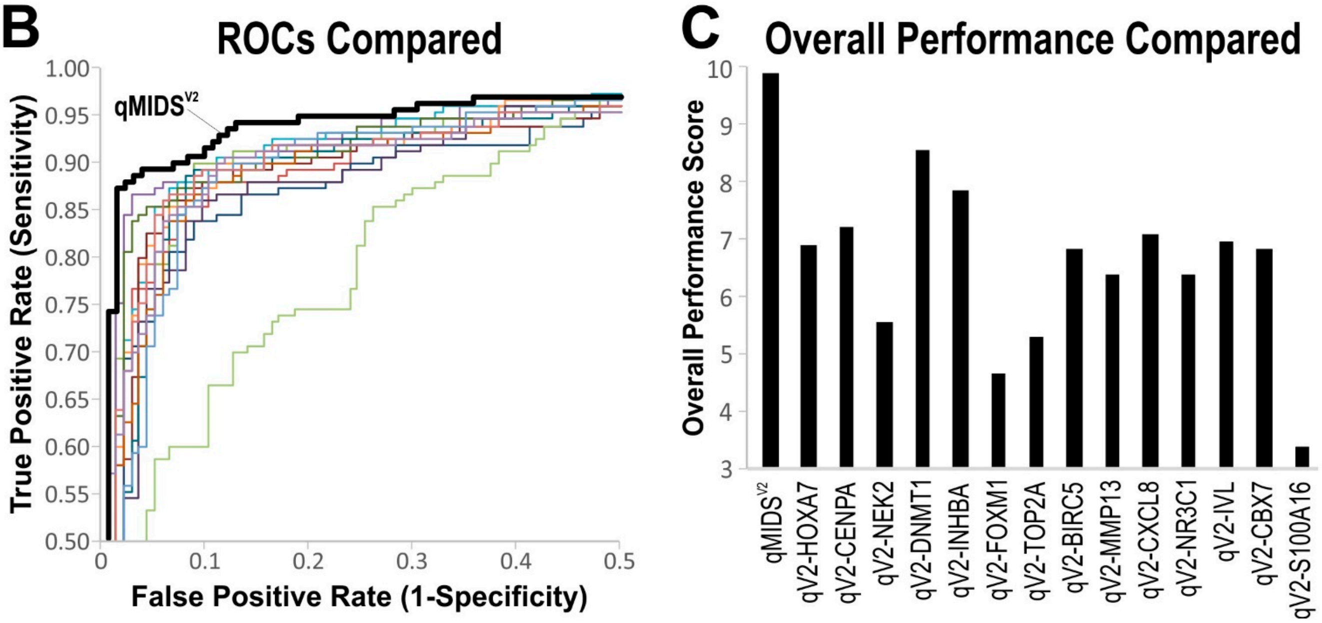

Figure S5. Effect of removing individual genes from the 14-target gene panel qMIDS^{V2} (qV2) on diagnostic test performance based on the UK patient cohort data. A, a table showing the diagnostic test performance data of removing each gene from qV2. A normalized overall performance scores were calculated to summarise the diagnostic performance for each gene removed. B, Data in panel A were subjected to ROC analysis for comparisons. AUC results (in %) for each curve are tabulated in panel A. C, Graphical representation of the overall performance scores calculated from data in panel A. TN, true negative; FN, false negative; FP, false positive; TP, true positive.
